# Physical Activity for the Treatment of Adolescent Depression: A Systematic Review and Meta-Analysis

**DOI:** 10.3389/fphys.2020.00185

**Published:** 2020-03-19

**Authors:** Max Oberste, Marie Medele, Florian Javelle, Heidrun Lioba Wunram, Daniel Walter, Wilhelm Bloch, Stephan Bender, Oliver Fricke, Niklas Joisten, David Walzik, Nicola Großheinrich, Philipp Zimmer

**Affiliations:** ^1^Department of Molecular and Cellular Sport Medicine, Institute of Cardiovascular Research and Sports Medicine, German Sport University Cologne, Cologne, Germany; ^2^Department of Child and Adolescent Psychiatry and Psychotherapy, Medical Faculty, University of Cologne, Cologne, Germany; ^3^Children's Hospital, Medical Faculty, University of Cologne, Cologne, Germany; ^4^School of Child and Adolescent Cognitive Behavior Therapy (AKiP) at the University Hospital Cologne, Cologne, Germany; ^5^Chair of Child and Adolescent Psychiatry, Witten/Herdecke University, Department of Child and Adolescent Psychiatry, Psychotherapy and Child Neurology, Gemeinschaftskrankenhaus Herdecke, Herdecke, Germany; ^6^Department of Social Sciences, Catholic University of Applied Science of North Rhine – Westphalia, Cologne, Germany; ^7^Department of “Performance and Health (Sports Medicine)”, Institute of Sport and Sport Science, Technical University Dortmund, Dortmund, Germany

**Keywords:** adolescent, depression, physical activity, meta-analysis, moderator

## Abstract

**Background:** A noticeable proportion of adolescents with depression do not respond to guideline recommended treatment options. This systematic review and meta-analysis investigated the effectiveness of physical activity interventions as an alternative or complementary treatment for adolescents (12–18 years) with depression. The characteristics of the physical activity treatment that were most effective in reducing symptoms in adolescents with depression and the impact of methodological shortcomings in the existing research were also examined.

**Methods:** Medline, PsycINFO, SPORTDiscus, ProQuest, and CENTRAL were searched for eligible records. Effect size estimates were pooled based on the application of a random-effects model. Potential moderation by physical activity characteristics (i.e., intensity, type, context, and time frame) and methodological features (i.e., type of control group and diagnostic tool to identify depression at baseline) was investigated by means of subgroup analyses and meta-regressions. The certainty of evidence was assessed by the Grading of Recommendations, Assessment, Development, and Evaluation (GRADE) approach. The primary outcome was the antidepressant effect of physical activity at postintervention measurement time point. As secondary outcomes, the sustainability of effects after the end of physical activity treatment and the acceptability of physical activity treatments were assessed. Overall, 10 studies were included in the qualitative synthesis and 9 studies involving 431 patients were included in the quantitative synthesis.

**Results:** A moderate, significant antidepressant effect of physical activity was found (Hedges' g = −0.47, 95% CI = −0.71 to −0.24). Heterogeneity was small (T^2^ = 0.0313, *I*^2^ = 27%, *p* = 0.18). However, the certainty of evidence was downgraded to low because the included studies contained serious methodological limitations. Moderator analyses revealed that session intensity significantly moderated the antidepressant effect of physical activity. Moreover, noticeably smaller effect sizes were found in studies that used non-physical activity sham treatments as control treatments (e.g., playing board games), compared to studies that used no control group treatments. Only three studies assessed the sustainability of effects after the end of physical activity treatment. The results suggest that the antidepressant effects further increase after the end of physical activity interventions. There was no significant difference in dropout risk between the physical activity and control groups.

**Conclusions:** This review suggests that physical activity is effective in treating depression in adolescents. Physical activity sessions should be at least moderately intense [rate of perceived exertion (RPE) between 11 and 13] to be effective. Furthermore, our results suggest that physical activity treatments are well accepted. However, the low methodological quality in included studies might have led to effect overestimation. Therefore, more studies with higher methodological quality are needed to confirm the recommendation for physical activity treatments in adolescents with depression.

## Introduction

The term adolescence refers to the developmental phase between childhood and adulthood (Hopkins, 2014). During this phase of major physical, psychological, and social changes, rates of depression increase dramatically (Thapar et al., 2012; The National Institute of Mental Health, 2017), with 3 to 6% of adolescents meeting the full criteria of major depressive disorder (Satcher, 2000; Bettge et al., 2008). Both early identification and effective treatment of adolescent depression are particularly important because they can severely impede the personal and professional development of the individuals concerned (Jaffee et al., 2002; Hawton et al., 2012). If depression in adolescence is not successfully treated, there is often a recurrence of depressive episodes and chronicity in adulthood (Cook et al., 2009; Clayborne et al., 2019). Accordingly, the increased risk of suicide makes adolescent depression a potentially life-threatening condition (Hawton et al., 2012; Thapar et al., 2012).

In most countries, clinical guidelines for the treatment of adolescent depression recommend cognitive behavioral therapy (CBT) and interpersonal therapy as first-line psychotherapy. As pharmacotherapy, clinical guidelines recommend the selective serotonin reuptake inhibitor fluoxetine. Psychotherapy and pharmacotherapy can be conducted as monotherapy or as a combination of both (Emslie et al., 2002; Dolle and Schulte-Körne, 2013). Meta-analyses have shown that CBT (Klein et al., 2007; Zhou et al., 2015) and interpersonal therapy (Cuijpers et al., 2011; Zhou et al., 2015) have a moderate-to-large antidepressant effect in affected adolescents. However, psychotherapy can be inaccessible and expensive (Biddle et al., 2000; Asarnow et al., 2009). Regarding fluoxetine, a recent meta-analysis showed moderate improvements in depressive symptom severity in affected adolescents (Cipriani et al., 2016), but called the administration of pharmacotherapy into question. Pharmacotherapy entails possible side effects (e.g., Pinna, 2015; Cipriani et al., 2016). Fluoxetine and venlafaxine have been shown to be associated with an elevated risk of suicidal ideation and behavior in adolescents (Hammad et al., 2006; Bridge et al., 2007). Overall, a noticeable proportion of adolescents suffering from depression do not respond to CBT, interpersonal therapy, and/or pharmacotherapy and continue to experience depressive symptoms (March et al., 2007).

Recently, there is a growing interest in physical activity as an alternative or complementary treatment option for adolescent depression (Oberste et al., 2018; Wunram et al., 2018). For adults suffering from depression, meta-analyses have shown moderate-to-large antidepressant effects of physical activity treatments (Cooney et al., 2013; Schuch et al., 2016; Stubbs et al., 2016). Several existing meta-analyses have examined the effects of physical activity on depressive symptom severity in a range of populations, including depressed adolescents, healthy adolescents (Carter et al., 2016), adolescents suffering primarily from a condition other than depression (Larun et al., 2008), prepubertal children (Brown et al., 2013), and adults (Bailey et al., 2018). Subgroups analyses, conducted in these meta-analyses, support the conclusion that physical activity treatments are effective and moderately alleviate symptoms in adolescents with depression. The last literature review on the topic was published in 2016 (Bailey et al., 2018). However, there is currently a paucity of meta-analytic reviews, which investigate the antidepressant effects of physical activity treatments solely in depressed adolescents.

Existing meta-analyses do not provide information regarding the potential moderators of the antidepressant effect of physical activity in adolescents with depression. For example, it remains unclear what characteristics of physical activity treatment are most effective in reducing symptoms in adolescents with depression. Similarly, the influence of methodological shortcomings on the antidepressant effect of physical activity in adolescents with depression has yet to be examined.

In this systematic review and meta-analysis, we provide an update on the research on the antidepressant effects of physical activity treatments in adolescents suffering from depression, as well as the respective meta-analytic effect estimate. Moreover, we examine potential moderation by characteristics of the applied physical activity treatment (i.e., intensity, type, context, and time frame) and critical methodological features (i.e., type of control group and diagnostic tool to identify depression at baseline). As a main measure for the antidepressant effects of physical activity treatments, changes on continuous measures of depression symptom severity (i.e., “Children's Depression Inventory” and “Beck Depression Inventory”) are used. Additionally, we investigate the sustainability of effects after physical activity treatment termination, the effect of physical activity treatments on remission rates, and the acceptability of physical activity treatments in adolescents with depression.

## Methods

Before conducting this review, we registered the key features on PROSPERO (registration number: CRD 42019125905). The implementation of this systematic review and meta-analysis followed the methods described in the Cochrane Handbook of Systematic Reviews (Higgins and Green, 2011) and was completed in accordance with the “Preferred Reporting Items for Systematic Reviews and Meta-Analyses” (PRISMA) guidelines (Moher et al., 2009, 2015). The PRISMA checklist is provided in Supplementary Material 1 of this article.

### Trial Eligibility Criteria

The eligibility criteria for this review were based on the PICOS (P - Population, I - Intervention, C - Comparison, O - Outcome(s), S - Study Design) framework (Liberati et al., 2009). The previous review identified only a small number of studies that examined the effects of physical activity interventions on depression symptom severity in depressed adolescents (Bailey et al., 2018). Therefore, to gather a sufficient number of studies for meta-analysis and subgroups analyses, eligibility criteria for this review were defined rather broadly.

#### Population

Studies were eligible for this review if the mean age of participants was between 12 and 18 years old and comprised participants suffering from depression at baseline. Participants' depression at baseline had to be diagnosed via structured clinical interview, using either established diagnostic criteria or an established self-rating scale, on which participants had to reach a minimal threshold. Studies of participants suffering from additional health problems (e.g., obesity, diabetes, and cancer) were excluded.

#### Intervention

Studies that investigated the effect of a physical activity treatment on depressive symptom severity were included. “Physical activity” was understood to mean “[…] any bodily movement produced by the skeletal muscles that results in energy expenditure above resting levels,” as defined by the American College of Sports Medicine (Garber et al., 2011). Any physical activity intervention that fell under this definition fulfilled the intervention eligibility criterion of this review.

#### Comparison

Trials were eligible for this review if they compared the effects of physical activity with a control group treatment. This review examined physical activity treatments as alternative or complementary treatment for adolescent depression. Therefore, studies were not included if control group treatments were guideline-recommended therapy options for adolescent depression (e.g., CBT or fluoxetine). Studies were not included if control group treatment significantly increased the heart rate of the control group participants. This restriction was made to be able to attribute antidepressant effects of physical activity treatment to physiological adaptations to the physical activity.

#### Outcome

Studies use a variety of depression scales like, for example, the “Children's Depression Inventory” and the Beck Depression Inventory. Studies were included if they applied a continuous measure of depressive symptom severity at the postintervention measurement time point. Application of a continuous measure of depressive symptom severity was explicitly stated as eligibility criteria because depressive symptom severity was the primary outcome of this review.

#### Study Design

Not only randomized controlled studies (including cluster-randomized studies) but also non-randomized controlled trials were included in this review.

### Search Strategy

We searched the electronic databases “Medline,” “PsycINFO,” “SPORTDiscus,” “ProQuest Dissertations and Theses,” and the “Cochrane Central Register of Controlled Trials” from inception to January 29, 2019. Search terms were selected to capture a broad range of studies that could then be evaluated more extensively. To identify relevant studies, we used the following search algorithm:

(Depress* [Title/Abstract] OR “affective symptom*” [Title/Abstract] OR “affective disorder*” [Title/Abstract] OR “mood disorder*” [Title/Abstract]) AND (child* [Title/Abstract] OR infant* [Title/Abstract] OR adolesc* [Title/Abstract] OR pubert* [Title/Abstract] OR youth* [Title/Abstract] OR girl* [Title/Abstract] OR boy* [Title/Abstract] OR school* [Title/Abstract]) AND (exercis* [Title/Abstract] OR sport* [Title/Abstract] OR “physical activity” [Title/Abstract] OR “physical exertion” [Title/Abstract] OR “physical training” [Title/Abstract] OR “physical education” [Title/Abstract] OR running [Title/Abstract] OR jogging [Title/Abstract] OR walking [Title/Abstract] OR bicycling [Title/Abstract] OR swimming [Title/Abstract] OR “strength training” [Title/Abstract]). The detailed search strategy is provided in Supplementary Material 2 of this article.

The references of recent systematic reviews on this topic (Larun et al., 2008; Brown et al., 2013; Carter et al., 2016; Bailey et al., 2018), and the references of all included studies were also searched for eligible records. The literature search was independently conducted by two members of the review team (MO and MM). Any discrepancies between the two reviewers were resolved through consultation of the full text.

### Outcome Measures and Data Extraction

Information about the publication type, study design, sample characteristics, intervention details, control group details, and outcome data at both postintervention measurement and measurement time points with a delay after the end of the intervention was extracted. If an article did not report adequate data for meta-analysis or presented data only in a graph, the corresponding author of the respective study was conducted via email. If our data request was not met, but the article provided data in a graph, the software Web Plot Digitizer was used to obtain data from the figures (Rothagi, 2013; Tsafnat et al., 2014). If an included study compared the effect of several different physical activity treatments with a control treatment, and if these physical activity treatments did not differ in terms of the moderator variables under investigation in this review (see section Moderator Analysis below), data were combined in accordance with the procedures outlined in the Cochrane Handbook of Systematic Reviews (Higgins and Deeks, 2019). However, this applied only for one study. Mohammadi (Mohammadi, 2011) examined the effects of a tennis/badminton intervention and of a soccer/volleyball intervention against the same single control group. Because the groups were identical concerning the moderator variables tested in this review, the effects of both physical activity variants were pooled. Two members of the review team (MO and MM) independently conducted data extraction. Any inconsistencies between the two reviewers were resolved through consultation of the full text.

### Risk of Bias Assessment

The risk of bias within the included studies was assessed using the “Physiotherapy Evidence Database (PEDro) scale.” The PEDro scale consists of the following 11 items: (1) eligibility criteria, (2) random allocation, (3) concealed allocation, (4) baseline comparability, (5) blinding of assessors, (6) completeness of follow-up, (7) intention-to-treat analysis, (8) between group statistical comparisons, (9) point estimates and variability, (10) blinding of subjects, and (11) blinding of therapists. We rated each included study based on these 11 items. A study was rated “low risk of bias” for an item if the full text report of the study clearly stated that the item requirements were fulfilled. If the full text report of the study did not clearly describe that the item requirements were met, we rated the study “high risk of bias” for that item. Two members of the review team (MO and MM) rated the included studies using the PEDro scale. The initial level of agreement between raters was excellent [intraclass correlation coefficient (ICC) = 0.91]. Any inconsistencies between raters were resolved in consultation with the third author (PZ).

### Evaluation of the Certainty of Evidence

We rated the certainty of evidence, which contributed to the primary meta-analysis, using the “Grading of Recommendations, Assessment, Development, and Evaluation” (GRADE) framework. The GRADE framework categorizes the certainty of evidence into one of the following four categories: “high level of certainty,” “moderate level of certainty,” “low level of certainty,” and “very low level of certainty.” The certainty of evidence is downgraded if one or more of the following shortcomings are present: “limitations in study design,” “inconsistency,” “indirectness,” “imprecision,” and “publication bias” (Schünemann et al., 2013). Two members of the review team (MO and MM) independently conducted the GRADE rating. Any disagreements between raters were resolved in consultation with the third author (PZ).

### Moderator Analysis

We investigated if (1) characteristics of the applied physical activity treatments and/or (2) methodological features of the existing research moderated the antidepressant effect of physical activity treatments. Here, we describe how the effect size estimates from the included studies were categorized into subgroups for moderator analyses.

#### (1) Physical Activity Characteristics

*Intensity*. We conducted a subgroups analysis depending on whether or not studies applied low, moderate, or vigorous intense physical activity. Low, moderate, and vigorous intensities of physical activity sessions were operationalized according to the guidelines of Norton et al. (2010). Current recommendations suggest that adolescents should daily be physically active at moderate-to-vigorous intensity for at least 60 min (U.S. Department of Health Human Services, 2018). To examine if moderate-to-vigorous intensity exercise is more efficient than low intensity physical activity concerning its antidepressant effects, we also pooled moderate and vigorous physical activity and compared it to low intensity physical activity.

*Type of physical activity*. We conducted a subgroups analysis depending on whether or not the nature of the various physical activity treatments was game-based or standardized (i.e., not game-based).

*Time frame of physical activity treatment*. Based on meta-regression, we examined the moderating potential of four aspects of the time frame of the physical activity treatments: (i) session duration, (ii) number of sessions per week, (iii) number of weeks, and (iv) total extent of the physical activity treatment (calculated as the number of weeks multiplied by the number of sessions per week multiplied by the minutes per session).

*Context of the physical activity treatment*. We conducted a subgroups analysis depending on whether or not study participants received only physical activity/control group treatment or additional psychological therapies and/or pharmacotherapy.

#### (2) Methodological Features of Existing Research

*Type of control group*. We conducted a subgroups analysis depending on whether or not the studies used an active or a passive control group. We defined an active control group as follows: (i) participants receiving a sham treatment with psychosocial stimulation that is comparable to that of the physical activity group and (ii) sham treatment in the control group that does not lead to a significant increase in cardiovascular activity. We defined a control group as passive if the participants in that group did not receive such a sham treatment.

*Diagnostic tool to identify depression at baseline*. We conducted a subgroups analysis depending on whether or not studies diagnosed depression in adolescents at baseline, by means of a structured clinical interview or with a self-rating scale.

### Data Analysis

The primary meta-analysis examined the effect of physical activity treatments on depressive symptom severity in adolescents suffering from depression. To achieve this, the “bias corrected Hedges' g standardized mean difference” (SMD) between control group and physical activity group at postintervention was calculated for each study. The “R” software package was used with established guidelines (Schwarzer et al., 2015) to pool SMDs across studies using a random effects model. The interpretation of the size of pooled SMD followed Cohen's classification. Consequently, SMD values of 0.2, 0.5, and 0.8 were interpreted as small, moderate, and large effect sizes, respectively (Cohen, 2013). To allow for easier interpretation, the pooled SMD was also transferred back into the units of the Beck Depression Inventory, which is a widely used scale of depression symptom severity. To achieve this, we multiplied the pooled SMD with the standard deviation of the control group of that study, which had the largest control group sample (Carter et al., 2015). It should be noted that negative SMD values express an antidepressive effect of physical activity. We quantified “between study heterogeneity,” calculating the T^2^ and the Higgins' *I*^2^ statistic. Higgins' *I*^2^ values of 25, 50, and 75% were interpreted as a low, moderate, and large proportion of between-study heterogeneity, respectively (Higgins et al., 2003). Indication of small-study effect was investigated using funnel plot, Egger's test of the intercept, and Duval and Tweedie's trim and fill method (Egger et al., 1997; Duval and Tweedie, 2000; Sterne et al., 2011).

We conducted sensitivity analyses by excluding studies rated “high risk of bias” on PEDro scale items and, separately, by excluding studies only published as dissertation theses. Based on subgroups analyses and meta-regressions, we examined the moderation potential of the aforementioned variables. Treatment acceptability was analyzed using dropout as a surrogate and calculated as the difference in risk of dropout between physical activity and control group for each included study. Risk difference data were pooled using the Mantel–Haenszel method with random effects (Schwarzer et al., 2015).

## Results

### Selected Studies

**Figure 1:**
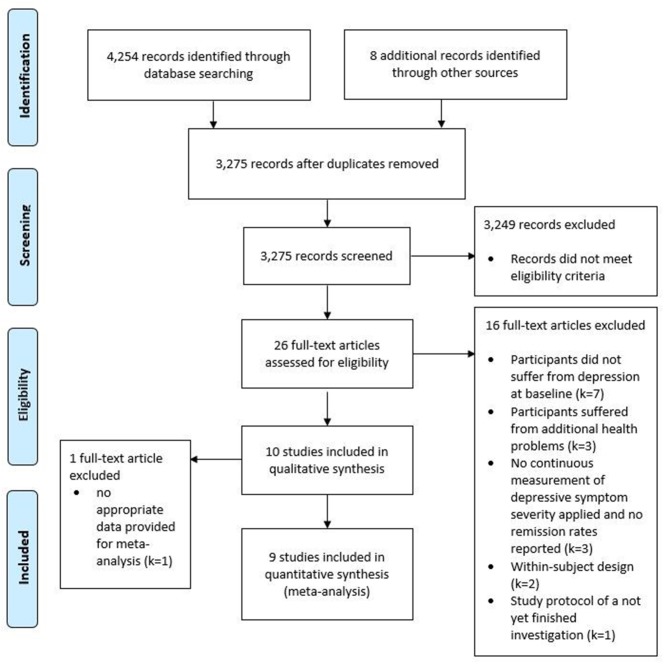
Flow chart of studies retrieved and screened according to the PRISMA guidelines.

The PRISMA diagram in Figure 1 provides an overview of the selection process. The final search result comprised 10 studies to be included in the qualitative synthesis of this review. However, 1 of the 10 studies (Moghaddam et al., 2012) did not provide sufficient data for meta-analysis and the corresponding author did not reply to our data request. Hence, nine studies, containing data from 431 adolescents suffering from depression, were included in the primary meta-analysis.

### Characteristics of Included Studies

Table 1 presents the characteristics of the studies that were included in the qualitative synthesis of this review.

**Table 1:** Characteristics of trials included in the qualitative synthesis of this review.

**References**	**Publication type**	**Study design**	**Sample description**	**Baseline diagnosis of depression**	**Exercise treatment**	**Control group treatment**	**Measurement of depression**
**Beffert (****1993****)**	• **Dissertation thesis**	• **Randomized controlled trial** • 8 weeks follow-up (no group comparison)	• **Non-clinical sample:** US American high school students • Additional treatments: participants received no other treatment for depression • Sample size: 26 20 (f)/6 (m) • Age: 12–16 years	• **Self-rating scale:** Reynolds Adolescent Depression Scale (score over 75)	• **6 weeks, 20 min 3 times/week walking–running program** • **Intensity: 60–85% of HR**_**max**_→ **vigorous** • **Dropouts: none out of 15** • Attendance: not reported • Organizational form: supervised group training	• **Passive control group**: waitlist-control • Dropouts: none out of 11	• **Self-rating scale:** Reynolds Adolescent Depression Scale
**Burrus (****1985****)**	• **Dissertation thesis**	• **Randomized controlled trial**	• **Nonclinical sample**: US American high school students • Additional treatments: participants received no other treatment for depression • Sample size: 45 27 (f)/18 (m) • Age: 15–18 years	• **Self-rating scale:** Depression Adjective Checklist (score above the 93rd percentile)	• **9 weeks, 45 min 4 times/week running training** • **Intensity: 75% HR**_**max**_ → **vigorous** • **Dropouts: none out of 14** • Attendance: not reported • Organizational form: supervised group training	• **Active control group:** classes in personal safety and first aid (4x/week for 9 weeks • Dropouts: none out of 15	• **Self-rating scale:** Multiscore Depression Inventory
					• **Nine weeks, 45 min 4 times/week weight training, softball, volleyball, and melon ball** • **Intensity: moderate** • **Dropouts: none out of 16** • Attendance: not reported • Organizational form: supervised group training		
**Carter et al. (****2015****)**	• **Published in peer-reviewed journal**	• **Randomized controlled trial** • 6 months follow-up	• **Clinical sample:** English adolescents receiving outpatient treatment for depression • Additional treatments: participants also received psychological therapies and, in rare cases, pharmacotherapy • Sample size: 87 68 (f)/19 (m) • Age: 15.4 ±.95 years	• **Self-rating scale:** Children's depression inventory 2 (score over 14)	• **6 weeks, 60 min 2 times/week circuit interval training with strengthening and aerobic exercises** • **Intensity: Mean heart rate was approximately 50% of HR**_**max**_ **and mean RPE was 10** → **low** • **Dropouts: 8 out of 44** • Attendance: Experimental group participants attended 70% of sessions • Organizational form: supervised group training	• **Passive control group:** treatment as usual • Dropouts: 14 out of 43	• **Self-rating scale:** Children's depression inventory 2
**Hughes et al. (****2013****)**	• **Published in peer-reviewed journal**	• **Randomized controlled trial** • 6 and 12 months follow-up (only remission rates reported)	• **Clinical sample:** US American adolescents receiving treatment for depression • Additional treatments: participants also received psychological therapies and/or pharmacotherapy • Sample size: 33 24 (f)/9 (m) • Age: 17 ± 2.34 years	• **Clinical interview:** DSM-IV-R criteria of Major Depressive Disorder	• **12 weeks, 35 min 3 times/week cycle ergometer training** **+** **home-based preferred physical activity** • **Intensity:** **>12 kilocalories/kilogram/week** → **vigorous** • **Dropouts: 2 out of 16** • Attendance: 77% of sessions • Organizational form: supervised group training	• **Active control group:** stretching and low-intense activities • Dropouts: 2 out of 14	• **Observer-rating scale:** Childhood Depression Rating Scale–Revised • Remission rates
**Jeong et al. (****2005****)**	• **Published in peer-reviewed journal**	• **Randomized controlled trial**	• **Non-clinical sample**: Korean middle school students • Additional treatments: participants received no other treatment for depression • Sample size: 40 (f) • Age: 16 years (SD not reported)	• **Self-rating scale:** Beck Depression Inventory (cut-off not further described)	• **12 weeks, 45 min 3 times/week Dance Movement therapy** • **Intensity: low** • **Dropouts: none out of 20** • Attendance: not reported • Organizational form: supervised group training	• **Passive control group:** no specific treatment • Dropouts: none out of 20	• **Self-reporting scale:** Depression scale of the Symptom Checklist-90-Revised
**Kanner (****1990****)**	• **Dissertation thesis**	• **Randomized controlled trial**	• **Clinical sample**: US American inpatients at a psychiatric treatment center • Additional treatments: participants also received psychological therapies and/or pharmacotherapy • Sample size: 68 28 (f)/40 (m) • Age: 13.63 ± 2.58 years	• **Self-rating scale:** Children's depression inventory 2 (score over 14)	• **6 weeks, 60 min 3 times/week cycle ergometer training** • **Intensity:** ** <60% HR**_**max**_→ **low** • **Dropouts: 5 out of 22** • Attendance: not reported • Organizational form: supervised group training	• **Active control group:** board games and pool while supervised • Dropouts**:** 7 out of 23	• **Self-rating scale:** Children's depression inventory 2
					• **6 weeks, 60 min 3 times/week cycle ergometer training** • **Intensity: 70–85% HRmax vigorous** • **Dropouts: 3 out of 23** • Attendance: not reported • Organizational form: supervised group training		
**Moghaddam et al. (****2012****)**	• **Published in peer-reviewed journal**	• **Randomized controlled trial**	• **Non-clinical sample**: Iranian high school students • Additional treatments: not reported • Sample size: 60 (m) • Age is not reported	• **Self-rating scale:** Beck Depression Inventory (threshold not further described)	• **12 weeks, 90 min 2 times/week swimming** • **Intensity: moderate** • **Dropouts: not reported** • Attendance: not reported • Organizational form: not reported	• **Passive control group:** no treatment • Dropouts: not reported	• **Self-rating scale:** Beck Depression Inventory
					• **12 weeks, 90 min 2 times/week track and field** • **Intensity: vigorous** • **Dropouts: not reported** • Attendance: not reported • Organizational form: not reported		
					• **12 weeks, 90 min 2 times/week football** • **Intensity: moderate** • **Dropouts: not reported** • Attendance: not reported • Organizational form: not reported		
**Mohammadi (****2011****)**	• **Published in peer-reviewed journal**	• **Randomized controlled trial**	• **Non-clinical sample**: Iranian high school students • Additional treatments: participants received no other treatment for depression • Sample size: 100 (gender distribution not reported) • Age is not reported	• **Self-rating scale:** Beck Depression Inventory (threshold not further described)	• **8 weeks, 75 min 3 times/week soccer and volleyball** • **Intensity: moderate** • **Dropouts: none out of 40** • Attendance: not reported • Organizational form: supervised group training	• **Passive control group:** no treatment • Dropouts: none out of 20	• **Self-rating scale:** Beck Depression Inventory
					• **8 weeks, 75 min 3 times/week table tennis or badminton** • **Intensity: moderate** • Dropouts: none out of 40 • Attendance: not reported • Organizational form: supervised group training		
**Dabidy Roshan et al. (****2011****)**	• **Published in peer-reviewed journal**	• **Randomized controlled trial**	• **Non-clinical sample**: Iranian high school students • Additional treatments: participants received no other treatment for depression • Sample size: 24 (f) • Age: 16.87 ±.91 years	• **Clinical interview:** DSM-IV_TR criteria of Major Depressive Disorder	• **6 weeks, 70 min 3 times/week aqua running** • **Intensity: 60–70% of HR**_**max**_→ **moderate** • **Dropouts: none out of 12** • Attendance: not reported • Organizational form: supervised group training	• **Passive control group**: no treatment • Dropouts: none out of 12	• **Observer-rating scale:** Hamilton Rating Scale for Depression
**Wunram et al. (****2018****)**	• **Published in peer-reviewed journal**	• **Non-randomized controlled trial** • 2 and 4 months follow-up	• **Clinical sample:** German patients at a psychiatric treatment center • Additional treatments: psychotherapy, art therapy, music therapy, and pharmacotherapy • Sample size: 64 46 (f)/18 (m) • Age: 15.9 ± 1.1 years	• **Clinical interview:** DSM-IV and ICD-10 criteria of Major Depressive Disorder	• **6 weeks, 30 min 3–5 times/week whole-body vibration strength training** • **Intensity: moderate** • **Dropouts: 3 out of 21** • Attendance: On average, patients participated in 22.1 ± 3.7 sessions • Organizational form: supervised group training	• **Passive control group:** treatment as usual • Dropouts: 6 out of 23	• **Self-rating scale:** Depression Inventory for Children and Adolescents • Remission rates
					• **6 weeks, 30 min. 3–5 times/week cycle ergometer training** • **Intensity: 3 min at 40%, 6 min at 50%, 3 min at 70–80%, 6 min at 50%, 3 min at 70–80%, and 6 min at 50% of individual VO2peak** → **vigorous** • **Dropouts: 3 out of 20** • Attendance: On average, patients participated in 23.5 ± 2.5 sessions • Organizational form: supervised group training		

*f female, m male, HR_max_ maximal heart rate, RPE rate of perceived exertion, DSV IV Diagnostic and Statistical Manual of Mental Disorders, 4th edition, ICD-10 10th revision of the International Statistical Classification of Diseases and Related Health Problems, VO_2_peak peak oxygen uptake*.

The 10 studies included in the qualitative analysis comprised 491 adolescents suffering from depression. The sample sizes of studies ranged from 24 (51) to 100 (39) (median = 52.5, interquartile range = 36.5). Two studies recruited only female participants (Jeong et al., 2005; Dabidy Roshan et al., 2011), while one study recruited only male participants (Moghaddam et al., 2012). In total, studies recruited approximately twice as many female adolescents with depression as male. The mean age of participants in the included studies ranged from 13.63 to 17 years old.

In 6 out of the 10 studies, participants received only the physical activity treatment for their depression. All of these studies were conducted in a school environment (Burrus, 1985; Beffert, 1993; Jeong et al., 2005; Dabidy Roshan et al., 2011; Mohammadi, 2011; Moghaddam et al., 2012). In the remaining four studies, participants received psychological therapies and/or pharmacotherapy in addition to physical activity/control group treatment. These studies were conducted in clinical contexts (Basen-Engquist et al., 2013; Hughes et al., 2013; Carter et al., 2015; Wunram et al., 2018). One study did not report details about additional treatments (Moghaddam et al., 2012).

The types of applied physical activity varied widely across studies. Three studies used cycling on an ergometer (Kanner, 1990; Hughes et al., 2013; Wunram et al., 2018). Three studies used physical activity games (e.g., football, badminton, volleyball) (Burrus, 1985; Mohammadi, 2011; Moghaddam et al., 2012). Two studies used walking/running (Burrus, 1985; Beffert, 1993). One study used circuit interval training (Carter et al., 2015), one dancing (Hughes et al., 2013), one swimming (Moghaddam et al., 2012), one track and field (Moghaddam et al., 2012), one aqua running (Dabidy Roshan et al., 2011), and one strength training on a whole-body vibration platform (Wunram et al., 2018).

### Risk of Bias

**Figure 2:**
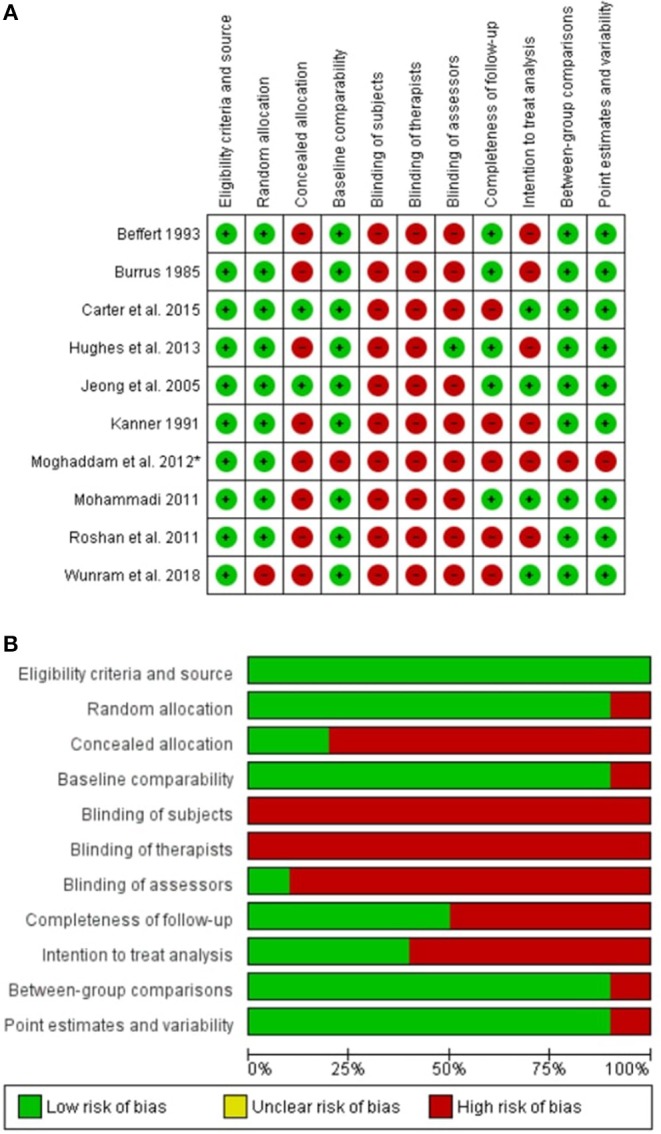
Risk of bias **(A)** within each study and **(B)** across studies (*study not included into meta-analysis as no data was provided).

All included studies described the source of recruitment of subjects and eligibility criteria for participants. Concerning the random allocation of participants into study groups, the nine included randomized controlled trials explicitly stated that allocation was random (Burrus, 1985; Kanner, 1990; Beffert, 1993; Jeong et al., 2005; Dabidy Roshan et al., 2011; Mohammadi, 2011; Moghaddam et al., 2012; Hughes et al., 2013; Carter et al., 2015). Random allocation was clearly not fulfilled in the one non-randomized controlled trial that was included in this review (Wunram et al., 2018). Group allocation was concealed in two studies (Jeong et al., 2005; Carter et al., 2015). Nine studies compared the data of treatment groups at baseline. One study fulfilled the criteria for blinding of assessors (Hughes et al., 2013). In five studies (Burrus, 1985; Beffert, 1993; Jeong et al., 2005; Mohammadi, 2011; Hughes et al., 2013), at least 85% of the participants gave data at the postintervention measurement time point (“completeness of follow-up”). Four studies (Jeong et al., 2005; Mohammadi, 2011; Carter et al., 2015; Wunram et al., 2018) conducted an intention-to-treat analysis. One study (Moghaddam et al., 2012) did not provide between group comparisons, as well as point estimates and measures of variability. Figure 2A summarizes the risk of bias within each study. Figure 2B summarizes the risk of bias across studies. None of the included studies provided blinding of subjects or blinding of therapists.

### Results of Primary Meta-Analysis

**Figure 3:**
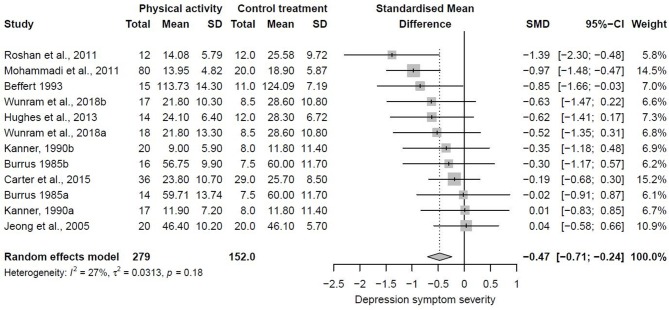
Forest plot of studies included in the primary meta-analysis.

As indicated above, nine out of the 10 studies provided data for the primary meta-analysis involving data of 431 patients. The nine studies included in the primary meta-analysis provided 12 effect size estimates due to the fact that three of the nine studies utilized multiple physical activity treatment groups. After pooling effect size estimates, a significant moderate antidepressant effect of physical activity treatments was found (Hedges' g = −0.47, 95% CI = −0.71 to −0.24). Heterogeneity was small (T^2^ = 0.0313, *I*^2^ = 27%, *p* = 0.18). This antidepressant effect corresponds to an average improvement of 3.99 points (95% CI = −6.06 to −2.04) in the Beck Depression Inventory (we used the standard deviation of the control group of included studies that had the largest sample size Carter et al., 2015 for back-transformation of pooled SMD). Figure 3 shows the forest plot of the primary meta-analysis.

### Small-Study Effect

**Figure 4:**
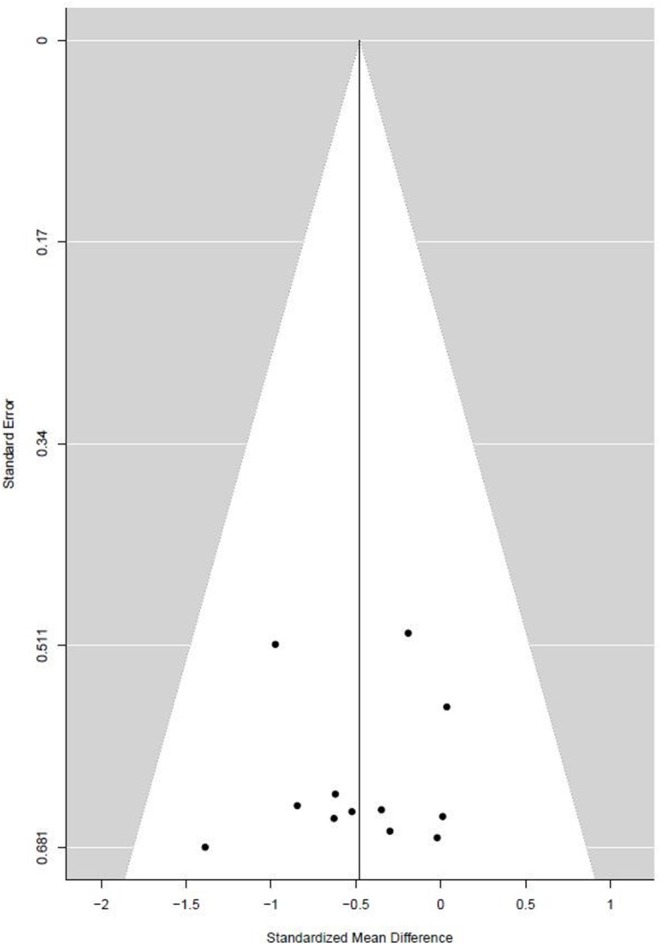
Funnel plot of the effect size estimates of physical activity treatments on depressive symptom severity in depressed adolescents against standard error.

The funnel plot, presented in Figure 4, shows the SMD of each individual study against its own precision (standard error). Visual inspection of the funnel plot does not reveal obvious asymmetry. The statistical testing of small-study effect using Egger's regression did not reach statistical significance (Egger's intercept = −0.24, *p* = 0.88). Duval and Tweedie's trim and fill analysis identified no additional studies on the right side of the funnel plot (SE = 0.16).

### Sensitivity Analysis

Outliers are commonly defined as the SMD of a study that has a confidence interval that does not overlap with the confidence interval of the pooled effect (Viechtbauer and Cheung, 2010). The forest plot in Figure 3 shows that this was not the case for any of the included studies. Therefore, sensitivity analysis by excluding outliers was not necessary.

One or more included studies were rated “high risk of bias” for the PEDro scale items “random allocation,” “concealed allocation,” “blinding of assessors,” “completeness of follow-up,” and “intention-to-treat analysis.” When the only non-randomized study (Wunram et al., 2018) was excluded from analysis, the aggregated effect did not change and heterogeneity increased compared to initial analysis (k = 10, Hedges' g = −0.46, 95% CI = −0.74 to −0.17, *p* = 0.0018, T^2^ = 0.0754, *I*^2^ = 39.6%). When the effect size estimates of studies that were rated “high-risk of bias” on the item “completeness of follow-up” were excluded, the pooled effect only marginally changed and heterogeneity increased compared to the initial meta-analysis (k = 6, Hedges' g = −0.5, 95% CI = −0.81 to −0.18, *p* = 0.0022, T^2^ = 0.0293, *I*^2^ = 39.6%). A similar result was achieved when the effect size estimates from studies rated “high-risk of bias” for “intention-to-treat analysis” were excluded (k = 5, Hedges' g = −0.45, 95% CI = −0.77 to −0.13, *p* = 0.0063, T^2^ = 0.0347, *I*^2^ = 48.1%).

We were not able to conduct a sensitivity analysis by excluding studies rated “high risk of bias” on the items “concealed allocation” (Jeong et al., 2005; Carter et al., 2015) and “blinding of assessors” (Hughes et al., 2013) as only two studies were rated “low-risk of bias” with regards to “concealed allocation” (Jeong et al., 2005; Carter et al., 2015) and only one study had “low-risk of bias” on “blinding of assessors” (Hughes et al., 2013). When the three studies, which published their results only in form of dissertation theses, were excluded from analysis, the antidepressant effect of physical activity became slightly higher but the heterogeneity increased (k = 7, Hedges' g = −0.56, 95% CI = −0.90 to −0.23, *p* = 0.0011, T^2^ = 0.0839, *I*^2^ = 47.7%). The results of all sensitivity analyses are summarized in Table 2.

**Table 2:** Sensitivity analysis.

**Sensitivity analyses**	**k**	***N***	**Hedges' g**	**95% CI**	***p*-value**	**Heterogeneity**
Primary meta-analysis	12	431	−0.47	−0.71 to −0.24	*p* < 0.0001	Q = 15.06, df = 11, *p* = 0.18, T^2^ = 0.0313, *I*^2^ = 27%
• Random allocation: low-risk of bias	10	379	−0.46	−0.74 to −0.17	*p* = 0.0018	Q = 14.9, df = 9, *p* = 0.09, T^2^ = 0.0754, *I*^2^ = 39.6%
• Concealed allocation: low-risk of bias^a^	2	105	−0.10	−0.49 to −0.28	–	–
• Blinding of assessors: low-risk of bias^b^	1	26	−0.62	−0.1.41 to 0.17	–	–
• Completeness of follow-up: low-risk of bias	6	237	−0.50	−0.83 to −0.18	*p* = 0.0022	Q = 8.28, df = 5, *p* = 0.14, T^2^ = 0.0293, *I^2^*= 39.6%
• Intention-to treat analysis: low risk of bias	5	257	−0.45	−0.77 to −0.13	*p* = 0.0063	Q = 7.7, df = 4, *p* = 0.1, T^2^ = 0.0347, *I*^2^ = 48.1%
• Only peer-reviewed articles	7	308	−0.56	−0.90 to −0.23	*p* = 0.0011	Q = 11.48, df = 6, *p* = 0.07, T^2^ = 0.0839, *I*^2^ = 47.7%

*k number of effect size estimates, N number of participants, Hedges' g pooled effect size estimate, CI confidence interval*.

a *The two included studies rated low-risk of bias for “concealed allocation” are Carter et al. (2015) and Jeong et al. (2005)*.

b *The only included study rated low-risk of bias for “blinding of assessors” is Hughes et al. (2013)*.

### Moderator Analysis

The description of how effect sizes estimates from the included studies were categorized into subgroups is provided in section *Moderator Analysis*. A summary of our moderator analysis is presented in Table 3.

**Table 3:** Moderator analysis based on the primary meta-analysis.

**Sensitivity analyses**	**k**	***N***	**Hedges' g**	**95% CI**	***p*-value**	**Heterogeneity**	**Test for subgroup difference/Meta regression**
Primary meta-analysis	12	431	−0.47	−0.71 to −0.24	*p* < 0.0001	Q = 15.06, df = 11, *p* = 0.18, T^2^ = 0.0313, *I^2^* = 27%	
**Methodological features:**							
**Type of control treatment**							
• Active control group	5	124	−0.27	−0.65	*p* = 0.1597	Q = 1.53, df = 4, *p* = 0.82, T^2^ = 0, *I*^2^ = 0%	Q-between = 1.52, df = 1, *p* = 0.22
• Passive control group	7	307	−0.59	to 0.11−0.93 to −0.25	*p* = 0008	Q = 11.95, df = 6, *p* = 0.06, T^2^=0.0902, I^2^=49.8%	
**Diagnostic tool at baseline**							
• Structured clinical interview	4	102	−0.76	−1.18 to −0.34	*p* = 0.0004	Q = 2.36, df = 3, *p* = 0.5, T^2^ = 0, *I^2^* = 0%	Q-between = 2.43, df = 1, *p* = 0.12
• Self-rating scale	8	329	−0.38	−0.62 to −0.13	*p* = 0.0024	Q = 10.28, df = 7, *p* = 0.17, T^2^ = 0.0035, *I^2^* = 31.9%	
**PA characteristics:**							
Intensity							
• Low	3	130	−0.08	−0.43 to 0.27	*p* = 0.6363	Q = 0.38, df = 2, *p* = 0.83, T^2^ = 0, *I*^2^=0%	Q-between = 6.72, df = 2, *p* = 0.03
• Moderate	4	174	−0.82	−1.27 to −0.36	*p* = 0.0004	Q = 3.7, df = 3, *p* = 0.30, T^2^ = 0.0695, *I*^2^ = 18.9%	
• Vigorous	5	127	−0.51	−0.88 to −0.14	*p* = 0.0074	Q = 2.11, df = 4, *p* = 0.72, T^2^ = 0, *I*^2^=0%	
**Intensity_2**							
• Low	3	130	−0.08	−0.43 to 0.27	*p* = 0.6363	Q = 0.38, df = 2, *p* = 0.83, T^2^=0, *I^2^* = 0%	Q-between = 7.27, df = 1, *p* = 0.007
• Moderate to vigorous	9	301	−0.68	−0.94 to −0.42	*p* < 0.0001	Q = 7.41, df = 8, *p* = 0.49, T^2^ = 0, *I*^2^=0%	
**Type of activity**							
• Standardized PA	9	267	−0.46	−0.73 to −0.18	*p* = 0.0011	Q = 8.55, df = 8, *p* = 0.38, T^2^ = 0.0217, *I^2^* = 6.5%	Q-between = 0, df = 1, *p* = 0.99
• PA embedded in games	3	164	−0.45	−0.1.02 to 0.12	*p* = 0.1184	Q = 6.39, df = 2, *p* = 0.04, T^2^ = 0.1428, *I*^2^ = 68.7%	
**Context**							
• PA in addition to psychological and/or pharmacotherapy	6	196	−0.34	−0.64 to −0.05	*p* = 0.0232	Q = 2.14, df = 5, *p* = 0.83, T^2^= 0, *I^2^* = 0%	Q-between = 0.75, df = 1, *p* = 0.39
• Only PA	6	235	−0.58	−1.03 to −0.13	*p* = 0.0063	Q = 11.45, df = 5, *p* = 0.04, T^2^ = 0.1699, *I^2^* = 56.3%	
**Time frame of PA treatment**							
• Session duration	–	–	–	–	–	–	Q-moderation = 0.35, df = 1, *p* = 0.56, *R*^2^ = 0%
• Number of sessions per week	–	–	–	–	–	–	Q-moderation = 0.04, df = 1, *p* = 0.85, *R*^2^ = 0%
• Number of weeks	–	–	–	–	–	–	Q-moderation = 0.24, df = 1, *p* = 0.63, *R*^2^ = 0%
• Total extent	–	–	–	–	–	–	Q-moderation = 0.001, df = 1, *p* = 0.98, *R*^2^ = 0%

*k number of effect size estimates, N number of participants, Hedges' g pooled effect size estimate, CI*.

*confidence interval, PA physical activity, PA physical activity treatment*.

#### (1) Physical Activity Characteristics

*Intensity*. Subgroups analysis revealed a significant difference between aggregated effect sizes of studies that applied physical activity at low, moderate, or vigorous intensity (Q-between = 6.72, df = 2, *p* = 0.03). Pooling of data from studies that applied low intensity physical activity resulted in a very small average effect size (k = 3, Hedges' g = −0.08, 95% CI = −0.43 to 0.27, *p* = 0.83, T^2^ = 0.0, *I*^2^ = 0%). Pooling of data from studies that applied moderate physical activity resulted in a large effect size (k = 4, Hedges' g = −0.82, 95% CI = −1.27 to −0.36, *p* = 0.30, T^2^ = 0.0695, *I*^2^ = 18.9%). Pooling of data from studies that applied vigorous physical activity treatments resulted in a moderate effect size (k = 5, Hedges' g = −0.51, 95% CI = −0.88 to −0.14, *p* = 0.72, T^2^ =0.0, *I*^2^ = 0%).

The comparison of effect sizes from studies that applied moderate or vigorous intensity physical activity to the effect sizes from studies that applied low intensity physical activity also reached statistical significance (Q-between = 7.27, df = 1, *p* = 0.007). Pooling of data from studies that applied moderate or vigorous intensity physical activity resulted in a moderate-to-large effect size (k = 9, Hedges' g = −0.68, 95% CI = −0.94 to −0.42, *p* ≤ 0.001, T^2^ = 0.0, *I*^2^ = 0%). Pooling of data from studies that applied low intensity physical activity resulted in a very small effect size (k = 3, Hedges' g = −0.08, 95% CI = −0.43 to 0.27, T^2^ = .0, I^2^ = 0%).

*Type of physical activity treatment*. Subgroups analysis revealed almost identical aggregated effect sizes from studies that used game-based physical activity (k = 3, Hedges' g = −0.45, 95% CI = −1.02 to 0.12, *p* = 0.12, T^2^ = 0.1428, *I*^2^ = 68.7%) and studies that applied a standardized physical activity treatment without game character (k = 9, Hedges' g = −0.46, 95% CI = −0.73 to −0.18, *p* = 0.001, T^2^ = 0.0217, *I*^2^ = 6.5%) (Q-between = 0, df = 1, *p* = 0.99).

*Context of the physical activity treatment*. Subgroups analysis revealed a small-to-moderate antidepressant effect aggregated from studies that applied physical activity/control group treatment in addition to psychological therapies and/or pharmacotherapy (clinical context) (k = 6, Hedges' g = −0.34, 95% CI = −0.64 to −0.05, *p* = 0.0232, T^2^ = 0, *I*^2^ = 0%). The effect aggregated from studies that only applied physical activity/control group treatment (school environment) was moderate to large (k = 6, Hedges' g = −0.58, 95% CI = −1.03 to −0.13, *p* = 0.0063, T^2^ = 0.1699, *I*^2^= 56.3%). The difference between the effect sizes of subgroups did not reach statistical significance (Q-between = 0.75, df = 1, *p* = 0.39).

*Time frame of physical activity treatment*. Meta-regression analyses revealed no relationship between any aspect of the time frame of the physical activity and antidepressant effects (session duration: Q-moderation = 0.35, df = 1, *p* = 0.56, *R*^2^ = 0%, number of sessions per week: Q-moderation = 0.04, df = 1, *p* = 0.85, *R*^2^ = 0%, number of weeks: Q-moderation = 0.24, df = 1, *p* = 0.63, *R*^2^ = 0%, total extent: Q-moderation = 0.01, df = 1, *p* = 0.98, *R*^2^ = 0%.

#### (2) Methodological Features of Existing Research

*Type of control group*. Pooling of data from studies that compared physical activity with an active control treatment resulted in a small antidepressant effect (k = 5, Hedges' g = −0.27, 95% CI = −0.65 to 0.11, *p* = 0.16, T^2^ = 0.0, *I*^2^ = 0%). Pooling of data from studies that used a passive control treatment resulted in a moderate-to-large antidepressant effect (k = 7, Hedges' g = −0.59, 95% CI = −0.93 to −0.25, *p* = 0.001, T^2^ = 0.0902, *I*^2^ = 49.8%). The difference between the effect sizes of subgroups did not reach statistical significance (Q-between = 1.52, df = 1, *p* = 0.22).

*Diagnostic tool to identify depression at baseline*. Data aggregated from studies that diagnosed depression at baseline using a structured clinical interview resulted in a large effect (k = 4, Hedges' g = −0.76, 95% CI = −1.18 to −0.34, *p* = 0.001, T^2^ = 0, *I*^2^ = 0%). Data pooled from studies that used a self-rating scale for baseline diagnosis of depression resulted in a small-to-moderate effect (k = 8, Hedges' g = −0.38, 95% CI = −0.62 to −0.13, *p* = 0.002, T^2^ = 0.0035, *I*^2^ = 31.9%). The difference between effect size of subgroups did not reach statistical significance (Q-between = 2.43, df = 1, *p* = 0.12).

### Analysis of the Sustainability of the Antidepressant Effects of Physical Activity

Only 3 out of the 10 studies, which were included in this review, reported data from measurement time points that were conducted after postintervention measurement time point. These data were not analyzed quantitatively due to too low statistical test power. Here, we describe the results of the three included studies that reported data from measurement time point after postintervention measurement time point.

Only two included studies, the study from Carter and colleagues and the study from Carter et al. (2015) and Wunram et al. (2018), captured continuous measures of depressive symptom severity at measurement time points after postintervention. Moreover, Hughes et al. (2013) reported only remission rates 14 weeks after the end of physical activity intervention.

At postintervention, Carter et al. (2015) reported a small antidepressant effect of physical activity compared to a control condition (Hedges' g = −0.19, 95% CI = −0.68 to 0.30). After 6 months without further physical activity treatment, this antidepressant effect approached almost a moderate level (Hedges' g = 0.39, 95% CI = −1.00 to 0.22). Wunram et al. (2018) reported moderate antidepressant effects for whole-body vibration strength training (Hedges' g = −0.52, 95% CI = −1.35 to 0.31) and for ergometer training (Hedges' g = −0.63, 95% CI = −1.47 to 0.22) at the postintervention measurement. Five months after the end of the physical activity treatment, this effect was large for both intervention types (whole-body vibration strength training: Hedges' g = −0.94, 95% CI = −1.78 to −0.11, ergometer training: Hedges' g = −0.91, 95% CI = −1.72 to −0.09). In the study by Hughes et al. (2013), at the postintervention measurement, 86% of the physical activity group participants no longer showed clinical signs of depression, while there was a remission rate of 50% in the control group. Fourteen weeks after the end of physical activity treatment, all physical activity group participants showed complete remission. This result remained stable until 40 weeks after the end of physical activity treatment. In the control group, 70% of participants showed complete remission at 14 weeks after the end of control treatment and 88% of participants at 40 weeks after the end of control treatment.

### Analysis of the Effects of Physical Activity Interventions on Remission Rate

Only 2 (Hughes et al., 2013; Wunram et al., 2018) out of the 10 studies, which were included in this review, measured the remission rates to evaluate the antidepressant effects of their physical activity treatment in adolescents with depression. This was the study from Wunram et al. (2018) and the study from Hughes et al. (2013). Remission rate data of the two studies was not analyzed quantitatively due to too low statistical test power. Here, we describe the results of the two studies that reported remission rate data qualitatively.

The study by Wunram et al. (2018) reported that, at postintervention, 29.9% of ergometer group participants, 50% of whole-body vibration strength training, and 17.6% of control group participants met criteria for remission. At 5-month follow-up, remission rates increased to 71.4, 61.5, and 25%, respectively. The remission rate data from the study by Hughes et al. (2013) is presented above (see section Analysis of Sustainability of the Anti-Depressant Effects of Physical Activity).

### Analysis of Acceptability of Physical Activity Treatment

All studies that were included in quantitative analysis reported dropouts during the course of treatment. On average, the dropout rate in physical activity groups was 7.01% (95% CI = 6.06 to 7.96). In control groups, the average dropout rate was 11.49% (95% CI = 9.37 to 13.6). We found no significant difference between the dropout risk in the physical activity groups and control groups (k = 9, RD = −0.02, 95% CI = −0.07 to 0.03, *p* = 0.40).

### Analysis of Adverse Events

Two studies reported monitoring of adverse events (Hughes et al., 2013; Wunram et al., 2018). In both of these studies, neither adverse events nor serious adverse events were reported to have a relationship with physical activity.

### Certainty of Evidence

We rated the certainty of evidence that contributed to the primary meta-analysis as low. Concerning “study design,” we identified serious limitations and downgraded the certainty of evidence by two levels. We did not downgrade the certainty of evidence for “inconsistency,” “indirectness,” “imprecision,” or “publication bias.” We provide a detailed description of the GRADE rating in Supplementary Materials 3, 4 in this article.

## Discussion

This meta-analysis is the first to investigate the effects of physical activity treatments on depressive symptom severity solely in adolescents with depression. A moderate antidepressant effect of physical activity in comparison to control treatments was found, with a confidence interval that ranged from a small to a large effect. This antidepressant effect is clinically important as it exceeds the current definition of minimal clinical important difference (Hiroe et al., 2005).

The size of the antidepressant effect of physical activity treatments, found here, is comparable to the size of the antidepressant effects that have been reported for treatments that clinical guidelines recommend for adolescent depression (Klein et al., 2007; Cuijpers et al., 2011; Zhou et al., 2015; Cipriani et al., 2016). However, unlike pharmacotherapy, physical activity seems largely free of side effects. Neither adverse nor serious adverse events associated with physical activity treatments were reported. In fact, physical activity has additional, positive effects on somatic pathologies, such as obesity and diabetes, which are themselves often associated with depressed mood and low self-esteem (Sjöberg et al., 2005; Crow et al., 2006).

However, the promotion of physical activity as treatment for adolescent depression should be adopted with some caution. The certainty of evidence was rated as low, and the studies that we included into this meta-analysis show methodological shortcomings. Therefore, we cannot rule out that we overestimated the effect of physical activity treatments on depression symptom severity due to selection and/or detection bias in the included studies. More studies of a higher methodological quality are needed to increase the strength of any recommendation for physical activity treatments in adolescents with depression.

The findings of our primary meta-analysis confirm the results of subgroups analyses from former meta-analyses, on adolescents with depression, which investigated the antidepressant effect of physical activity in more comprehensive populations (Larun et al., 2008; Brown et al., 2013; Carter et al., 2016; Bailey et al., 2018). However, beyond the pooled effect of physical activity treatments on depressive symptom severity, in adolescents with depression, we also provide information about potential methodological and physical activity moderators. Our subgroups analysis of the type of control group treatment revealed a noticeable difference between studies that used non-physical activity sham treatments as control treatments (e.g., playing board games), compared to studies that used no control group treatments. The effect aggregated from studies that used non-physical activity sham treatments as control treatments was much smaller than the effect aggregated from studies that used no control treatment. The small number of studies included in this review and its small statistical test power may explain why the distinct difference between the applied types of control treatments did not reach statistical significance. More pronounced effects are found in studies with no control treatments, compared to studies with non-physical activity sham treatments, which suggest the existence of placebo effects in included studies with passive control groups (Stothart et al., 2014; Lindheimer et al., 2015). It is plausible that the additional attention that physical activity group participants received in studies with no control group treatment led to the overestimation of the antidepressant effect found in this meta-analysis (Lindheimer et al., 2015). More studies that investigate the antidepressant effect of physical activity treatments in adolescents with depression compared to non-physical activity sham treatments are needed.

We found that studies using a structured clinical interview at study intake achieved larger antidepressant effects than studies that used self-rating scales. The small number of studies included in this review and its small statistical test power might explain the lack of statistical significance between the effects of the subgroups. Clinical interviews are the gold standard in diagnosis of depression in adolescents (Haugen et al., 2016). At the same time, the sensitivity of most self-rating scales aiming to identify depression among adolescents is not above 75% (Salle et al., 2012). Thus, the use of clinical interviews, compared to the use of self-reports, is likely to reduce the proportion of false-positive participant diagnoses in the included studies. Participants that suffer from depression clearly benefit more from an antidepressant treatment than misdiagnosed participants do. We recommend that future studies use structured clinical interviews, conducted by blinded experts, to check if participants are eligible for study participation.

Our review indicates that the intensity of the physical activity treatment is a major moderator. Subgroups analysis showed almost no antidepressant effect for low intensity physical activity, a large effect for moderate intensity physical activity, and a moderate effect for vigorous intensity physical activity. If moderate and vigorous intensity physical activity treatments were pooled to moderate to vigorous, the advantageous effect compared to low intensity physical activity treatments became even more significant. The finding that at least moderate intensity physical activity is needed to alleviate depressive symptoms might reflect the intensity-dependent response of physiological processes to physical activity. Several neurobiological mechanisms, which are discussed as mediators of the antidepressant effects of physical activity within the scientific community, show strong associations with physical activity intensity. These include, for example, hypothalamic–pituitary–adrenal axis activity (Duclos and Tabarin, 2016), neurotrophin (Dishman, 2006; Carek et al., 2011) and growth factor expression (Krogh et al., 2014), oxidative stress, inflammatory markers (Nabkasorn et al., 2006; Carek et al., 2011; Byrne et al., 2013), and alterations of the kynurenine pathway (Agudelo et al., 2014).

The antidepressant effect does not differ between studies that used physical activity embedded in games, and studies that used standardized physical activity without game character. It seems that the additional psychosocial stimulation of physical activity games did not contribute additionally to the antidepressant effect of physical activity treatments. This result is surprising as a recent meta-analysis showed small, beneficial effects of play therapy (Zhou et al., 2015).

We found that the effect aggregated from studies in a clinical setting was noticeably smaller than the effect aggregated from studies in a school environment. In this review, all studies in clinical setting conducted physical activity/control group treatments in addition to psychological therapies and/or pharmacotherapy while included studies in the school environment conducted only physical activity/control group treatments. The fact that the antidepressant effect of physical activity remained in the presence of psychological therapies and/or pharmacotherapy suggests that physical activity may effectively complement the existing treatments that clinical guidelines recommend for the therapy of adolescent depression.

None of the aspects of the time frame of the physical activity treatment under investigation moderated the antidepressant effect of physical activity in adolescents with depression. This may mean that the antidepressant effect of physical activity quickly becomes apparent. The minimum session duration (20 min), minimum frequency per week (2 times per week), minimum number of weeks (6 weeks), and minimum total extent (360 min) applied in included studies may be sufficient to achieve effects. However, the small amount of data on this question makes this inference quite speculative. More studies that investigate the influence of the time frame of physical activity treatments are needed in order to make physical activity treatments as efficient as possible.

The low number of studies that applied measurement time points that were conducted after postintervention did not allow for a quantitative analysis of the sustainability of physical activity antidepressant effects. However, the three studies with measurement time points that were conducted after postintervention indicate further development of the antidepressant effects of physical activity intervention between the end of treatment and following measurement time points. Physical activity treatments seem to alleviate depressive symptom severity long after the ending of the respective treatment. One possible reason for this might be that participants maintain regular physical activity after the end of the treatment. However, to the best of our knowledge, data regarding changes of the physical activity behavior due to physical activity treatment in adolescents with depression are lacking until today.

Finally, yet importantly, our results show that adolescents with depression tolerate physical activity very well. In fact, the difference between dropout risk in physical activity groups and in control groups, found here, is lower than those reported for psychological therapies (de Haan et al., 2013) and for pharmacotherapy with selective serotonin reuptake inhibitors (Hetrick et al., 2012). This finding clearly indicates the feasibility of incorporating physical activity treatment in existing clinical treatment programs for adolescent depression.

## Conclusion

This meta-analysis indicates that physical activity treatments may be effective in alleviating depressive symptom severity among adolescents suffering from depression, either as an alternative or additional treatment option. However, our findings should be interpreted with caution. Concerns about the methodological quality of existing research restrict the certainty of evidence. Moreover, our results raise concerns about placebo effects in existing research. Moderator analyses indicate that physical activity should be of at least moderate intensity to alleviate depressive symptoms in affected adolescents. At the same time, embedding physical activity in games does not seem to be a prerequisite for antidepressant effects. Aspects of the time frame of physical activity treatments were not associated with the antidepressant effect of physical activity. Future studies should use structured interviews, conducted by blinded experts, to check if participants are eligible for study participation. Finally, we found that adolescents with depression tolerate physical activity very well.

## Author Contributions

MO, MM, NJ, and DW performed material preparation and data collection. MO, MM, FJ, WB, and PZ performed the data analyses. HL, DW, SB, OF, and NG provided background knowledge about psychopharmaceutic and psychotherapeutic treatment of adolescent depression. MO and MM wrote the first draft of the manuscript. All authors contributed to the study conception and design, commented on previous versions of the manuscript, and read and approved the final manuscript.

### Conflict of Interest

The authors declare that the research was conducted in the absence of any commercial or financial relationships that could be construed as a potential conflict of interest.
