# Supplementary material for: Physical Activity for the Treatment of Adolescent Depression: A Systematic Review and Meta-Analysis
Source: Front Physiol. 2020 Mar 19;11:185. doi: 10.3389/fphys.2020.00185 (PMC7096373; doi:10.3389/fphys.2020.00185)
Supplement: Supplementary file 2 [file Table_2.DOCX]

ESM2. Search terms.

| **Population** | child* [Title/Abstract] OR infant* [Title/Abstract] OR adolesc* [Title/Abstract] OR pubert* [Title/Abstract] OR youth* [Title/Abstract] OR girl* [Title/Abstract] OR boy* [Title/Abstract] OR school* [Title/Abstract]) |
| --- | --- |
| **AND** | |
| **Outcome** | depress* [Title/Abstract] OR “affective symptom*” [Title/Abstract] OR “affective disorder*” [Title/Abstract] OR “mood disorder*” [Title/Abstract]) |
| **AND** | |
| **Intervention** | exercis* [Title/Abstract] OR sport* [Title/Abstract] OR “physical activity” [Title/Abstract] OR “physical exertion” [Title/Abstract] OR “physical training” [Title/Abstract] OR “physical education” [Title/Abstract] OR running [Title/Abstract] OR jogging [Title/Abstract] OR walking [Title/Abstract] OR bicycling [Title/Abstract] OR swimming [Title/Abstract] OR “strength training” [Title/Abstract] |
| **Study design limits** | Above search limited to: English language and German language, only human trials |
