# Supplementary material for: Physical Activity for the Treatment of Adolescent Depression: A Systematic Review and Meta-Analysis
Source: Front Physiol. 2020 Mar 19;11:185. doi: 10.3389/fphys.2020.00185 (PMC7096373; doi:10.3389/fphys.2020.00185)
Supplement: Supplementary file 3 [file Table_3.DOCX]

ESM3. GRADE rating assessing the certainty of the evidence that contributed to the summary effect of physical activity on depressive symptoms calculated in the primary meta-analysis.

| RCT level evidence: HIGH ⊕⊕⊕⊕ | | |
| --- | --- | --- |
| **1. Limitation in study design (risk of bias):** Very serious limitations | **Action:** Consider downgrading two levels ⊖⊖ | **Reason:** We included randomized and non-randomized controlled trials into this review. Including not solely randomized controlled trials means, that certainty of evidence will automatically be downgraded for limitations in design (risk of bias) [1]. Moreover, seven out of nine trials included into the meta-analysis suffered from inadequate concealment of allocation (selection bias) and eight out of nine included trials suffered from unblinded assessment of outcome. In four out of nine included trials, less than 85% of participants gave data at post-intervention measurement. Five trials were rated high risk of bias because analyses were conducted as intention-to-treat.  - Lack of allocation concealment: 7/9  - Lack of blinding: 7/9  - Incompleteness of follow-up: 3/9, 1/9 unclear  - Selective outcome reporting: 4/9, 1/9 unclear  - Other limitations: One CT included (Wunram)  The proportion of information from studies at high risk of bias is sufficient to affect the interpretation of results. Plausible bias that seriously weakens confidence in the results. We downgraded the certainty of the evidence by two levels due to these study limitations. |
| **2. Inconsistency:** Unlikely | **Action:** do no downgrade | **Reasons:** Ninety-five percent confidence intervals of all trials included into the meta-analysis overlapped. All trial level 95% CIs overlapped. Moreover, the proportion of between-study heterogeneity (Higgins’ I^2^) was low according to Cochrane Handbook (I^2^=27%) [2] and not significant (*p* = 0.18).  We did not downgrade the certainty of evidence. |
| **3. Indirectness:** Unlikely | **Action:** do not downgrade | **Reason:** All included trials were relevant to the review question, no indirect comparators were used, all trials recruited adolescents with at least threshold levels of depressive symptoms, all reported depression symptoms as outcomes.  We did not downgrade the certainty of evidence |
| **4. Imprecision:** Unlikely | **Action:** do not downgrade | **Reason:** The total number of participants included into the meta-analysis was 431. That is more than the number of patients generated by a conventional sample size calculation for a single adequately powered trial. The effect size is larger than 0.2 standard deviations. Ninety-five percent confidence interval of summary effect does not cross the line of no effect. Ninety-five percent confidence interval of summary effect is relatively narrow with a lower level above .2 standard deviations.  We did not downgrade the certainty of evidence. |
| **5. Publication Bias**: Unlikely | **Action:** do not downgrade | **Reason:** The search strategy was not restricted to internationally peer-reviewed articles but also included dissertation theses. The funnel plot did indicate publication bias. Egger’s regression did not reach significance (Egger’s intercept= -.24, p=.88) and Duval and Tweedie’s trim and fill analysis suggested no additional studies on the right side (SE=.16)  We did not downgrade the certainty of evidence. |
| **Overall certainty of evidence rating:** Low ⊕⊕⊖⊖  **Interpretation**   \| Low ⊕⊕⊖⊖ \| Our confidence in the effect estimate is limited: The true effect may be substantially different from the estimate of the effect. \|  \| \| --- \| --- \| --- \| | | |

References

1. Balshem H, Helfand M, Schünemann HJ, Oxman AD, Kunz R, Brozek J, et al. GRADE guidelines: 3. Rating the quality of evidence. J Clin Epidemiol. 2011;64:401–6. doi:10.1016/j.jclinepi.2010.07.015.

2. Higgins JPT, Green S. Cochrane Handbook for Systematic Reviews of Interventions Version 5.1.0 [updated March 2011]. The Cochrane Collaboration; 2011. www.handbook.cochrane.org.
