# Supplementary material for: Physical Activity for the Treatment of Adolescent Depression: A Systematic Review and Meta-Analysis
Source: Front Physiol. 2020 Mar 19;11:185. doi: 10.3389/fphys.2020.00185 (PMC7096373; doi:10.3389/fphys.2020.00185)
Supplement: Supplementary file 4 [file Table_4.DOCX]

ESM4. Summary of Findings for the Main Outcome.

| **Physical Activity compared to control for adolescents suffering from depression**  **Population:** adolescents with depression  **Setting:** any setting  **Intervention:** Physical Activity  **Comparison:** active treatments not significantly stimulating cardiovascular system or passive treatments (including TAU) | | | | | |
| --- | --- | --- | --- | --- | --- |
| **Outcomes** | **No of Participants**  **(studies, effect sizes estimates)** | **Quality of the evidence**  **(GRADE)** | **Illustrative comparative risks (95% CI)** | | **Comments** |
|  |  | | **Assumed risk** | **Corresponding risk** |  |
|  |  |  | **Control** | **Exercise** |  |
| **Symptoms of depression**  Different scales  Post-intervention | 431  (9 studies, 12 effect sizes estimates) | to Low  ⊕⊕⊖⊖^1,2,3^ | The mean symptoms of depression in the control group was  **0** | The mean symptoms of depression in the intervention group was  **.48 standard deviations lower** (.71 to .24 lower) | SMD -.48 (95% CI: -.71 to - .24).  The effect sizes was interpreted as ‘moderate’ (*using Cohen’s rule of thumb*) |
| **^*^**T**he risk in the intervention group** (and its 95% CI) is based on the assumed risk in the comparison group and the **relative effect** of the intervention (and its 95% CI).  **CI:** Confidence interval | | | | | |
| GRADE Working Group grades of evidence  **High quality:** Further research is very unlikely to change our confidence in the estimate of effect.  **Moderate quality:** Further research is likely to have an important impact on our confidence in the estimate of effect and may change the estimate.  **Low quality:** Further research is very likely to have an important impact on our confidence in the estimate of effect and is likely to change the estimate.  **Very low quality:** We are very uncertain about the estimate. | | | | | |
| ^1^ Randomized and non-randomized trials included into the review  ^2^Seven out of nine included trials suffered from high risk of bias due to lack of concealment of participant allocation (selection bias).  ^3^Eight out of nine included trials suffered from high risk of bias due to lack of outcome assessor blinding | | | | | |
